# Social stigma against individuals with COVID-19: scale development and validation

**DOI:** 10.1080/21642850.2022.2155166

**Published:** 2022-12-28

**Authors:** Angga Wilandika, Nina Gartika, Salami Salami

**Affiliations:** Department of Nursing, Faculty of Health Sciences, Universitas Aisyiyah Bandung, West Java, Indonesia

**Keywords:** COVID-19, psychometrics, social stigma, stereotyping, discrimination

## Abstract

**Background:**

Social stigma toward individuals with COVID-19 is a public phenomenon that significantly impacts the prevention of this disease. The study aimed to develop and examine the scale of social stigma against people with COVID-19.

**Methods:**

A cross-sectional study was conducted from June to August 2021 using random sampling. Two hundred twenty-five people were involved in the study. All people are domiciled in Bandung Regency, West Java, Indonesia and have never been infected with COVID-19. The scale was designed based on the dimensional structure of social stigma and then evaluated the scale's psychometric properties.

**Result:**

The study found that instruments with 12 items had a content validity index of 1.0. Cronbach's alpha coefficient of 0.875 showed as satisfactory. Exploratory factor analysis was performed on the first sample (n = 100), and four factors were extracted from the exploratory factor analysis: ignorance/labelling, stereotype, separation, and discrimination. Following this, the confirmatory factor analysis in the remaining sample (n = 120) showed a good fit between the four-factor model and the theoretical model of social stigma.

**Conclusions:**

The social stigma scale has been determined to be valid and reliable. Health practitioners can use this scale to predict social stigma toward individuals with COVID-19 to develop better transmission prevention strategies and improved quality of care.

## Introduction

COVID-19, which has spread since December 2019, has influenced all life perspectives. Issues due to this disorder reach open well-being both physically and mentally. COVID-19 also raises far-reaching psychosocial impacts and concerns within the community (Abdelhafiz & Alorabi, 2020; Young et al., 2021). The problem raised by this illness stems from the illness's characteristics, which include a fast and massive transmission capacity that's troublesome to control. In addition, the infection is said to be new, so the treatment of this illness is still within the improvement arrangement. In addition to causing physical symptoms, this illness also causes various psychological effects such as fear, anxiety, stress, depression, sleep disorders, and other psychological disorders (Alimoradi et al., 2021; Alimoradi et al., 2022; Rajabimajd et al., 2021).

COVID-19 affects everyone, whether it's patients, the community, or even health workers who experience anxiety disorders that are increasingly developing into depression (Olashore et al., 2021; Sandya et al., 2022). Moreover, with no drug declared to cure COVID-19, efforts to combat the disease are focused on preventing its spread. Preventive actions include limiting social distance and making individuals more aware of implementing health protocols (Young et al., 2021). However, preventive transmission endeavours are carried out against individuals enduring from COVID-19 by being kept absent from sound individuals, interaction limitations, and conjointly isolated.

However, much news circulated through the media causes fears, stereotypes, and the emergence of stigma (Roy et al., 2021; Shetty K et al., 2021). This experience of stigma has become a tiring problem for people with COVID-19. They experience stigma from others when they find out the status of COVID-19. Experiences that arise such as fear of losing support from friends or neighbours and adverse attitude reactions toward them (Sangma et al., 2022). The emergence of stigma related to COVID-19 is caused by inadequate information about this disease. Inappropriate information related to the disease spreading in the community drives the emergence of diversity in the community. What is more, COVID-19 is a new disease, so the facts about this disease are still not well conveyed. Society is becoming increasingly difficult to distinguish between right and wrong information. Therefore, news and rumours are widely spread (Romer & Jamieson, 2020; Simonov et al., 2020). Social media could be a significant source of the emergence of the stigma of COVID-19 (Pennycook et al., 2020).

The social stigma against people with COVID-19 significantly impacts how we deal with the disease. The stigma of COVID-19 harms public health because it can cause the public to avoid examination and take precautions; moreover, this stigma severely impacts the mental health of those stigmatized (Bharadwaj et al., 2017; Islam et al., 2021). Thus, the prevention and treatment of stigma must be handled to prevent the occurrence of further implications of this social stigma.

Social stigma is the parameter of a psychosocial condition that can be assessed through a measurement. Handling social stigma must be based on appropriate, consistent, and reliable assessment standards. Therefore, a good instrument in measuring the phenomenon of social stigma against a condition such as social stigma in people with COVID-19 must be designed and have good validity. The study aims were to develop social stigma assessment instruments against people with COVID-19 and analyze the psychometric characteristics of the instrument.

## Methods

### Design and context

This research was conducted from June to August 2021. During this period, in the Bandung Regency, West Java Province, the government is imposing restrictions on emergency social activities (PPKM-Darurat) from 3 to 25 July 2021 based on the Regulation of the Minister of Home Affairs of the Republic of Indonesia. The increase in COVID-19 infections and social restrictions have raised public suspicion of the disease and the people who suffer from it. If not handled, this social stigma that appears in the community will make it challenging to handle COVID-19 completely. Therefore, a comprehensive measurement is needed to identify possible stigma. As a result, developing measuring tools to assess social stigma toward the individual with COVID-19 in society is essential.

### Development of the items

The development of the instrument is designed to measure social stigma toward the individual with COVID-19 in the community. The scale of social stigma against COVID-19 is adapted based on the stigma theory of Scheid & Brown (Scheid & Brown, 2010). Based on that theory, stigma is formed in four aspects: labelling, stereotype, separation, and discrimination. This social stigma theory is used because it corresponds to the conditions in which COVID-19 infection occurs in the community. The disease causes worry and fear due to incorrect assumptions and tendencies about attitudes and behaviours. So that, in the end, gives rise to negative marking, separation, to cause isolation or treatment to stay away from people affected by the disease.

Labelling is the first activity in stigmatizing someone. A person considered different because of the disease will be identified with a particular characteristic. After that, someone who is stigmatized will ignore the stigmatized person. The labelling aspect related to COVID-19 is associated with the origin of the disease, so people who come from this place will be considered different. One item leads to this explanation, “COVID-19 is a Chinese virus”. Labelling can also be associated with disease characteristics such as: “COVID-19 can be transmitted anytime, anywhere, and from anyone”, “People with COVID-19 should undergo isolation and be kept away from the general public”, and “People should be afraid and stay away from those who are sick from COVID-19”. Question items in this domain also refer to the assumption about the disease, such as “COVID-19 is a burden on society” and “COVID-19 is a punishment from God”.

The stereotype domain has five items. First, stereotype refers to a person's thoughts associated with negative characteristics. This belief is considered trustworthy by individuals who practice prejudice. For example, the stereotype can appear with the idea about the element of disease that should be avoided, such as items: “COVID-19 is a deadly contagious disease that exceeds other infectious diseases”, “When a person shows symptoms of COVID-19, he or she is suspected of COVID-19”, and “When a new person is travelling from a distant place, it should be checked for COVID-19”. In addition, the stereotyped domain can also be associated with a reluctance to interact because it makes someone uncomfortable. For example, there are two items: “People should be afraid and stay away from those who are sick from COVID-19” and “I won't visit a sick neighbour when I find out he has COVID-19”.

Separation means being away from and kept away if it is within the scope of stigma. People suffering from the disease will be shunned or expected to stay away from society. Even people who interact with sufferers are also targeted. Separation is measured through five items: “When a person shows symptoms of COVID-19, he or she should be immediately hospitalized”, “A person who dies from COVID-19 should not be buried in a public cemetery”, “Families of COVID-19 sufferers should not live in the community”, “People with COVID-19 are not eligible to live close to other neighbours”, and “People who are declared cured of COVID-19 should not gather with other communities”.

Discrimination is a domain related to aspects of behaviour or actions. Discrimination is measured in five items: “I am not willing to stay with people who have been declared cured of COVID-19”, “I will not go to the hospital for fear of contracting COVID-19”, “People who work in health services and close contact with COVID-19 patients should be isolated and kept away from the community”, “I am not pleased to help the needs of families with COVID-19 who are in self-quarantine”, and “Families of COVID-19 sufferers cannot transmit to the surrounding community”.

### Adaptation procedures

The adaptation procedure was conducted by three experts examining the 20-items draft scale. An expert from the University of ‘Aisyiyah Bandung and one expert from Gunung Jati Hospital, Cirebon, have expertise in the context of stigma against a disease. The validity of this content is to get input to the quality of the entire statement item so that it can measure what you want to measure. In addition, this test is done to determine the suitability of each item and whether the reader can easily understand it. Each expert comments on all items. Then assess the aspects of language, clarity of meaning, and conformity of the thing referred to in theory.

After the adaptation process for 20 self-administered items was carried out, a pilot study was also conducted on 30 adults. The participants involved in the pilot study were people aged 20–42 with a mean of 24.1 ± 5.1. Participants with male and female sex in the same proportion amounted to 15 people (50%). The educational level of the participants ranges from secondary education (80%) to higher education (20%). After filling in 20 question items, each participant gave their opinion about the ease of reading and understanding each item. They were also asked to provide input if there was a sentence structure for each item to be modified. The participants’ filling out the questionnaire also measured how long it took the questionnaire to be completed. After that, we met a final item. The pilot study data did not analyze further.

### Data collection procedures

Cross-sectional designs are applied in these studies. A total of 220 people were involved in the study. The inclusion criteria used in this study are 1) all citizens domiciled in Bandung Regency, West Java, Indonesia, 2) aged 19 - 55 years, and 3) have never been infected with COVID-19. Data retrieval uses random sampling techniques concerning inclusion criteria. Collecting data by distributing online questionnaires to respondents in the Bandung Regency area. The questionnaire was distributed by research assistants placed in six villages in the Bandung Regency, then spread to residents in the area. Residents willing to be involved will be given a link to the questionnaire that must be filled out.

The first sample (n = 100) was used to perform consistency reliability analysis (CRA) and the exploratory factor analysis (EFA), while the last sample (n = 120) was used to analyze confirmatory factor analysis (CFA). The sample size is considered sufficient to perform exploratory factor analysis. EFA can give reliable results for N well below 50, even in minor distortion (de Winter* et al., 2009). The data was analyzed using IBM 26.0 SPSS software.

### Assessment of psychometric properties

Psychometric characteristics are assessed by assessing each item's content validity index (CVI). An assessment of the content validity index is carried out to ensure the unity of the scale plan (Polit & Beck, 2006). CVI values are given by experts in the range of scores of 1–4 for each item. The scored accordingly: 1 = test not being relevant; 2 = somewhat relevant; 3 = quite relevant and; 4 = highly relevant. Grades 3 and 4 were considered acceptable. In studies involving panels of experts of five or fewer, CVI values are expected to reach 1.0 to ensure the validity of the contents. In addition, all experts are expected to approve that this instrument is suitable for use and relevant to describe the phenomenon to be measured (Kääriäinen et al., 2020).

The social stigma scale consists of 20 items on a 4-point Likert scale with a range of 1 point to strongly disagree until point 4 to strongly agree. All items are divided into four domains: five items labelling aspect, five items aspect stereotype, five items aspect separation, and five items aspect discrimination. Social stigma scores are done by summing the entire score on each item. Then the score was categorized into high and low levels.

Moreover, psychometric measures were conducted to determine validity, including scale reliability, EFA and CFA. A standard of reliability is the internal consistency resulting from a combination of consistency between variables on a scale. Measurements are made by examining each item's correlation, including the correlation of items with the number of all items. First, evaluate the correlation with individual items, such as the correlation between items. Results are acceptable when the correlation of items with total items is 0.30 or higher (Hair et al., 2014). The second measure is the reliability coefficient. Reliability coefficient measurements are also performed to assess the consistency of each item and the total scale. The reliability coefficient is acceptable if Cronbach's Alpha score must have a score of 0.70 or higher (Taber, 2018).

This study conducted Kaiser-Meyer-Olkin (KMO) and Bartlett's sphericity test to measure sampling adequacy. KMO values ⁣⁣between 0.70 and 0.79 are moderate, and values ⁣⁣between 0.60 and 0.69 are low. A KMO value of less than 0.60 indicates inadequate sampling and requires corrective action. An average KMO value > 0.60 is acceptable for a sample size of less than 100. In addition, the significant value of Bartlett's test < 0.05 shows that factor analysis may be worth the data set (Shrestha, 2021; Watkins, 2021).

The EFA was used to identify the relationship between variables in constructing a construct. EFA is used because the indicators made do not yet have a grouping hypothesis into the variables of the created scale. Extract factors using eigenvalues greater than 1.0 and principal components factor (PCF) and rules orthogonal rotation (Howard, 2016; Yong & Pearce, 2013). Factor loading values ⁣⁣equal to or greater than 0.3 are necessary for factor analysis (Howard, 2016). However, if 100 samples are used, the loading factor is 0.55 or greater (Hair et al., 2016).

These data studies continued for CFA. CFA assesses the relationship between the measured indicator and the latent variable or factor. CFA is needed to determine in advance all aspects that fit the model. These indices were used to assess the appropriate fit between the observed and theoretical models. These are considered an acceptable fit indices: the relative Chi-square index (χ2/df) < 5; the Root Mean Square Error of Approximation (RMSEA) < 0.10; the comparative fit index (CFI) and Tucker-Lewis index (TLI) > 0.90; Standardized Root Mean Squared Residual (SRMR) = 0.05-0.10; and Coefficient of Determination (CD) > 0.90 (Brown, 2015).

### Ethical clearance

Data related to the study were collected after obtaining approval from the Research Ethics Committee from Universitas Aisyiyah Bandung (No. 29/KEP.01/Unisa-Bandung/VI/2021). Informed consent was obtained from all individual participants included in the study. Before data collection, all respondents were given information about the nature of the study. The data collected were reported in general terms and did not involve any identifying data. All the data were kept confidential and securely held for the required time. The data were entered into a computerized database, and the use of a code protected the identity of the participants.

## Results

### Expert review about content validity

The social stigma scale of 20 items has a statement structure that can describe what you want to measure. All experts give a good assessment and agree with the dimensional structure of this scale. No items are omitted. The CVI assessment score on each item ranges from 3 to 4, so the CVI of 20 items reached a value of 1.0.

However, some items need to be added with explanatory sentences to understand better and not cause misinformation. This change as in point 20, there is the addition of the word “contagious” in describing the characteristics of COVID-19 disease. Point 15, a sentence change that shows a more stereotypical domain is “COVID-19 is a punishment from God”. In addition, in point 8, because there are similarities in meaning with the previous item, the sentence changes to “people who work in health services and close contact with COVID-19 patients should be isolated and kept away from the community”. After repairing and customizing this entire item, the last version is compiled. Thus, this scale that has been formed is tested and shows no difficulty in understanding all the grains on this instrument. Completion of this instrument takes about 15–20 min.

### Baseline characteristics of participants

The socio-demographics of participants are shown in Table 1. 220 people in the Bandung Regency, Indonesia, were involved in this study. Most people are between 19 and 55 years old, and most are male (58.6%). Most participating citizens are married (75.9%), with education as extensive as graduates from high school (50.5%). In addition, most citizens have worked (68.2%) either as private employees, civil servants, entrepreneurs, or workers.

**Table 1.** Socio-demographics of participants.

Demographics	Total(n = 220)	CRA and EFA (n = 100)	CFA (n = 120)
Age (Mean ± SD) Range 19–55 years	26.8 ± 8.3	26.4 ± 8.3	27.2 ± 8.2
*Gender*			
Male	129 (58.6)	58 (58.0)	71 (59.2)
Female	91 (41.4)	42 (42.0)	49 (40.8)
*Marital*			
Married	167 (75.9)	73 (73.0)	94 (78.3)
Not married	53 (24.1)	27 (27.0)	26 (21.7)
*Education*			
Higher education	111 (50.5)	59 (59.0)	52 (43.3)
Secondary education	109 (49.5)	41 (41.0)	68 (56.7)
*Occupation*			
Work	150 (68.2)	63 (60.0)	87 (72.5)
Does not work	70 (31.8)	37 (37.0)	33 (27.5)

Notes: CRA (consistency reliability analysis); EFA (exploratory factor analysis); CFA (confirmatory factor analysis).

### Reliability analysis

Table 2 shows data from the internal consistency reliability analysis, where four items were removed from the scale because it does not correlate with the total item score (correlation value < 0.3). Furthermore, the modification scale of 15 items is re-numbered. The scale's internal consistency with standardized Chronbach's alpha coefficient was 0.875. The correlation score of each item on the scale was between 0.395-0.743, which means it has a medium to a high standard.

**Table 2.** Chronbach’s α Analysis (n = 100).

Item	Original Scale			Modified Scale		
	Item-Test Corr.	Item-Rest Corr.	α	Item-Test Corr.	Item-Rest Corr.	α
*Separation*						
When a person shows symptoms of COVID-19, they should be immediately hospitalized.	0.511	0.424	0.813	0.486	0.382	0.873
A person who dies from COVID-19 should not be buried in a public cemetery.	0.742	0.689	0.799	0.743	0.687	0.860
Families of COVID-19 sufferers should not live in the community.	0.720	0.668	0.801	0.735	0.683	0.860
People with COVID-19 are not eligible to live close to other neighbours.	0.721	0.677	0.804	0.741	0.698	0.861
People who are declared cured of COVID-19 should not gather with other communities.	0.687	0.625	0.803	0.705	0.643	0.862
*Discrimination*						
I am not willing to stay with people who have been cured of COVID-19.	0.719	0.657	0.800	0.711	0.645	0.861
I will not go to the hospital for fear of contracting COVID-19.	0.528	0.443	0.812	0.541	0.452	0.871
People who work in health services and have close contact with COVID-19 patients should be isolated and kept away from the community	0.631	0.559	0.806	0.630	0.554	0.866
I am not pleased to help the needs of families with COVID-19 who are in self-quarantine.	0.396	0.333	0.818	0.395	0.329	0.875
Families of COVID-19 sufferers cannot transmit to the surrounding community.	0.171	0.073	0.830	Exclude		
*Labelling*						
COVID-19 is a Chinese virus.	0.455	0.372	0.816	0.508	0.426	0.872
COVID-19 can be transmitted anytime, anywhere, and from anyone.	0.039	−0.037	0.831	Exclude		
People with COVID-19 should undergo isolation and be kept away from the general public.	0.691	0.624	0.802	0.726	0.662	0.860
COVID-19 is a burden on society.	0.610	0.513	0.808	0.604	0.501	0.870
COVID-19 is a punishment from God	0.565	0.477	0.810	0.586	0.497	0.869
*Stereotype*						
People should be afraid and stay away from those sick from COVID-19.	0.444	0.347	0.817	0.463	0.363	0.875
When a person shows symptoms of COVID-19, they are suspected of COVID-19.	0.470	0.394	0.815	0.509	0.433	0.871
When a new person travels from a distant place, it should be checked for COVID-19.	0.127	0.015	0.835	Exclude		
I won't visit a sick neighbour when I find out he has COVID-19.	0.176	0.072	0.831	Exclude		
COVID-19 is a deadly contagious disease that exceeds other infectious diseases.	0.075	−0.029	0.835	Exclude		
*Total*			**0**.**823**			**0**.**875**

### Exploratory factor analysis

Table 3 shows the KMO coefficient value of 0.782, and Bartlett's result test was 513.656 (*p* < 0.001). These results indicate that the number of samples is appropriate and meets the requirements for EFA analysis. Meanwhile, Anti-Image Matrices show two items with a Measure of Sampling Adequacy (MSA) value < 0.55, and the item is removed. A total of 12 items were re-measured.

**Table 3.** Post estimation Factor of the Social Stigma Scale (n = 100).

Factor Postestimation Value	Total	EFA
*Kaiser-Meyer-Olkin coefficiency*	0.678	0.782
*Bartlett test*		
χ^2^	964.102	513.656
Degrees of freedom	190	66
*p*-value	0.001	0.001

**Figure 1.**
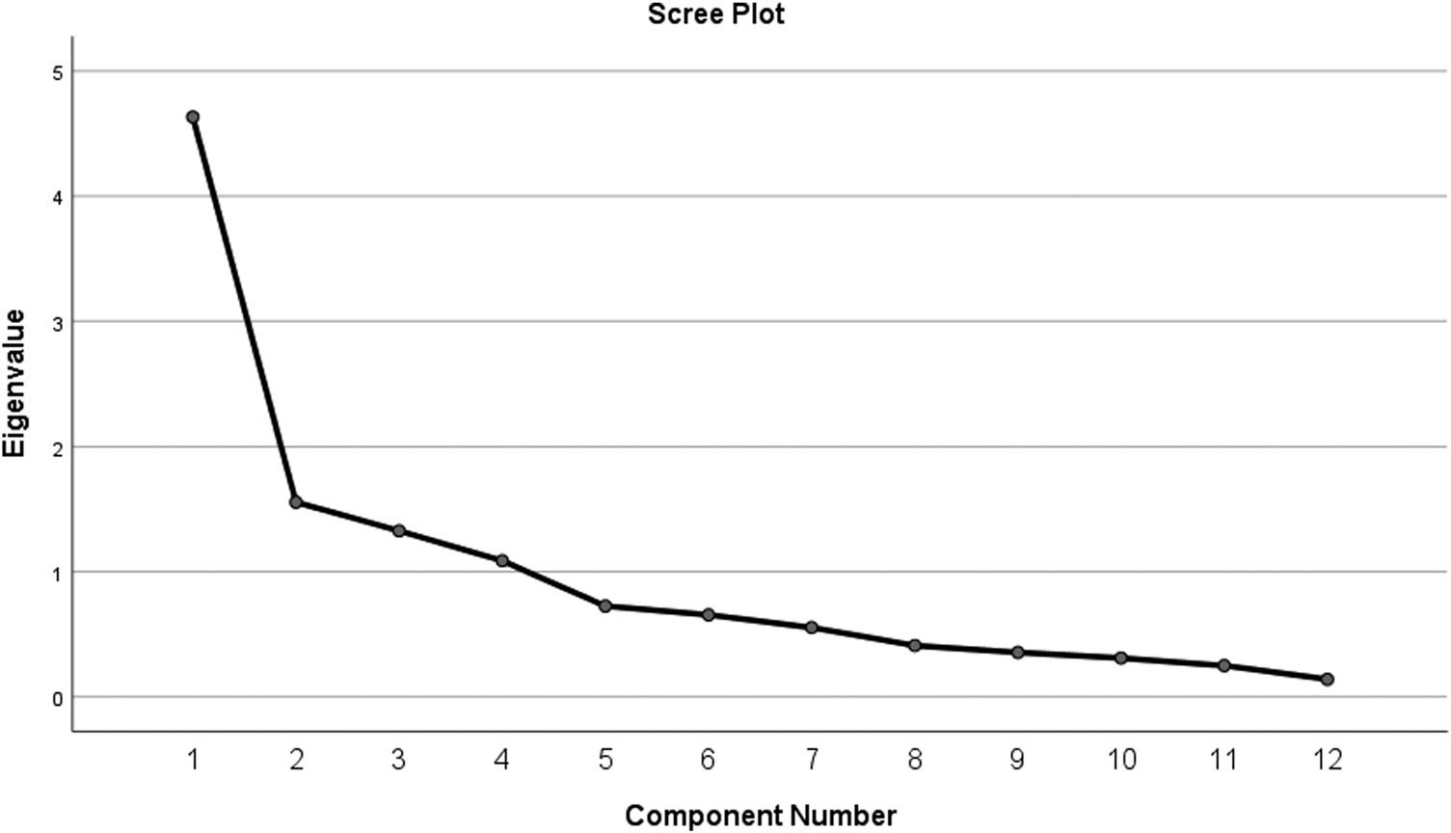
Scree plot of eigenvalues after EFA (n = 100).

The results of EFA showed four factors extracted from exploratory analysis with eigenvalues > 1 and loading factor > 0.5. The variance explained by the newly formed factor is 71.71% (Table 4). Similarly, the results of the scree plot examination were the same as those of the four forming factors (Figure 1). Based on the analysis factor formed separation factor (4 items), discrimination (4 items), stereotypes (2 items), and ignorance and labelling (2 items).

**Table 4.** EFA of the Social Stigma Scale (n = 100).

Item		Factor 1	Factor 2	Factor 3	Factor 4
1.	When a person shows symptoms of COVID-19, they should be immediately hospitalized.	0.706	−0.229	0.125	0.276
2.	Families of COVID-19 sufferers should not live in the community.	0.801	0.381	0.199	0.013
3.	People with COVID-19 are not eligible to live close to other neighbours.	0.800	0.357	0.095	0.098
4.	People who are declared cured of COVID-19 should not gather with other communities.	0.818	0.166	0.075	0.207
5.	I am not willing to stay with people who have been cured of COVID-19.	0.323	0.555	0.102	0.425
6.	I will not go to the hospital for fear of contracting COVID-19.	0.111	0.795	−0.015	0.114
7.	People who work in health services and have close contact with COVID-19 patients should be isolated and kept away from the community	0.187	0.751	−0.024	0.333
8.	I am not pleased to help the needs of families with COVID-19 who are in self-quarantine.	0.091	0.621	0.480	−0.249
9.	COVID-19 is a Chinese virus.	0.224	0.090	0.028	0.733
10.	People with COVID-19 should undergo isolation and be kept away from the general public.	0.118	0.374	0.304	0.696
11.	People should be afraid and stay away from those sick from COVID-19.	0.129	−0.181	0.781	0.417
12.	When a person shows symptoms of COVID-19, they are suspected of COVID-19.	0.179	0.173	0.850	0.030
Eigenvalue		4.633	1.556	1.327	1.088
Explain variance		38.61%	12.97%	11.06%	9.07%
*Cumulative explain variance*		**71.71%**			

Note: Factor 1 = separation; factor 2 = discrimination; factor 3 stereotypes; and factor 4 = ignorance/labelling.

### Confirmatory factor analysis

**Figure 2.**
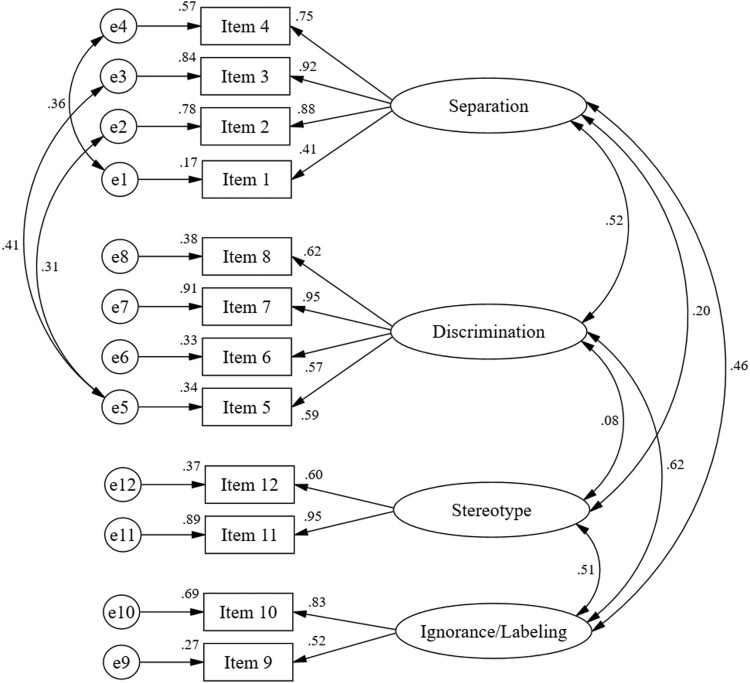
CFA for the Social Stigma Scale (n = 120).

As shown in Figure 2, data were analyzed from the second sample (n = 125), and model fits were found for four dimensions of social stigma against people with COVID-19. The four dimensions have a strong relationship with each other, with the standardized covariance value of each item ranging from 0.27–0.91. Factor loadings of 12 items were accepted, with 0.41-0.95. The multiple square correlations ranged from 0.164–0.911, which means that 50.0% had a high correlation (≥0.50), indicating that each adjusted factor contributed to the model.

The next stage was to modify the former model to get the best fit for the model. Model revised by adding covariations between items “When a person shows symptoms of COVID-19, he or she should be immediately hospitalized” and “People who are declared cured of COVID-19 should not gather with other communities”; “I am not willing to stay with people who have been cured of COVID-19” with “ Families of COVID-19 sufferers should not live in the community” and “ People with COVID-19 are not eligible to live close to other neighbours”. Good fit of the statistics obtained: χ^2^ /df = 1.67, RMSEA = 0.075, CFI = 0.948, TLI = 0.924, SRMR = 0.078, and CD = 0.174-0.894 (Table 5). Figure 2 shows the perfectly standardized revised four-factor model.

**Table 5.** Goodness-of-Fit Statistics.

Fit Statistic	Value	Expected Value
*Likelihood ratio test for model vs saturated comparison*		
χ^2^ /df	1.67	
*p*-values	0.003	>0.05
*Population error*		
RMSEA	0.075	<0.08
RMSEA 90% CI, lower bound	0.044	
RMSEA 90% CI, upper bound	0.104	<0.1
Probability RMSEA ≤ 0.05	0.085	
*Baseline comparisons*		
CFI	0.948	>0.9
TLI	0.924	Close to 1
*Size of residuals*		
SRMR	0.078	<0.08
CD	0.164–0.911	Close to 1

## Discussions

The final scale of the 12-items social stigma scale against people with COVID-19 was proved valid and reliable for measuring the level of social stigma in society. This scale showed good internal consistency reliability assessed using Cronbach's alpha coefficients. Four factors were extracted from the EFA with eigenvalues >1 and factor loading > 0.55, indicating that the model explained 71,71% of the determining variances, which is reasonable. In terms of construct validity, CFA showed that the model fitted the data well, including acceptable indices of CFI, TLI, RMSEA, SRMS, and CD.

The social stigma that occurs in society becomes essential to know. Measuring social stigma is an attempt to detect early the phenomena that lead to stigma. If this stigma is allowed, it can develop more severe and impact handling COVID-19 disease control. Therefore, stigmatization of diseases or people who experience COVID-19 has consequences of complicating disease control (Saiz et al., 2021). The government and other health sectors have also campaigned for various programs to prevent stigma (Ahorsu et al., 2020; Lin, 2020; Logie & Turan, 2020). However, most of the responses from the public still focus on preventing and avoiding diseases, such as distance restrictions and travel bans.

On the other hand, efforts to prevent COVID-19 disease trigger the emergence of stigma related to COVID-19 (Logie & Turan, 2020). Prevention of COVID-19 utilizes restrictions on everyone's distance, prohibition of leaving the house or travelling far, and quarantine for people affected by COVID-19, triggering community perceptions that can develop into the stigma. Therefore, it is necessary to develop appropriate strategies to improve disease prevention efforts and reduce stigma (Logie & Turan, 2020; Xu et al., 2021). This is so as not to cause conflict between disease control efforts and the impact it causes, primarily related to stigma.

The first step that can be done is to know how much stigmatization occurs. Thus, a measuring tool is needed to show the phenomenon. A viable and reliable instrument for measuring a flawed phenomenon is the first step in improving the condition. According to Sallis et al. (Sallis et al., 2000), developing standardized, reliable, and validated psychometric instruments is a crucial step in health promotion interventions that should be positioned early.

Information on societal stigma towards people with COVID-19 can be used to determine health requirements and handle relevant situations using suitable measuring instruments. If it is recognized how much stigma exists in the society, psychological and social problems associated with COVID-19 can be minimized. Based on the results of this study, the scale of social stigma against people with COVID-19 is an instrument used in assessing the phenomenon of stigma carried out by the community against people who have COVID-19. These measurements are essential to reduce the impact of this social stigma, especially on emerging ones.

Social stigma against people with COVID-19 is a psychological and social phenomenon surrounding the occurrence of labelling, prejudice, segregation, and discrimination against people with COVID-19 committed by the community. This social stigma against people with COVID-19 is multidimensional, covering the dimensions of knowledge, attitudes, and behaviour (Link & Phelan, 2001; Scheid & Brown, 2010; Wilandika & Sari, 2020). In this study, social stigma was formed from four aspects: labelling, stereotype, separation, and discrimination. All these aspects of walking can occur gradually or on their own and ultimately form a stigma.

Social stigma is characterized by the tagging or labelling of socially distinct or prominent characters from the rest of the majority. This form of labelling is also manifested in negative tagging applied by others to people's rebuttal differently due to a social condition or behaviour that eventually leads to being shunned (Link & Phelan, 2006). Labelling done in people with COVID-19 can be associated with the initial characteristics of the disease. The labelling, such as COVID-19, comes from China, a deadly virus and infection that can be transmitted anytime, anywhere, and from anyone. The labelling aspect of COVID-19 can also be attributed to the assumption that this disease becomes a burden on the community.

Each individual tends to distinguish one thing from another and label it accordingly (Huda, 2022). A habit forces one to determine the appropriate norms for socially selecting human differences. However, all citizens generally have this labelling practice, although these labelling practices vary according to the conditions, place, and time. The general pattern of labelling these differences is more likely to be associated with negative stereotypes (Link & Phelan, 2006).

Individuals who have “undesirable attributes” such as COVID-19 disease are considered to be different from the wider community and are considered separate from “us” (Huda, 2022). Stereotypical aspects on the scale of social stigma can be assessed through statements such as “COVID-19 sufferers are a punishment for people who disobey God”, or the notion that hospitals are the source of COVID-19 transmission, and the statement “families of COVID-19 sufferers can transmit to the surrounding community”.

All negative attributes in people with COVID-19 are identified based on the nature of the disease. These attributes are attached to the sufferer and give rise to stereotypes of people who have the disease. Even on some occasions, these attributes remain bound even though the person has been cured. This stereotypical aspect of social stigma arises from a person's or a group of people's beliefs toward another that is perceived to be different due to attributes considered wrong. As a result, those with these other attributes are perceived as disabled or polluted. The inevitable consequence of this negative labelling and stereotype is the loss of the dignity status of the individual, degrading one's position to the lowest level in the eyes of others (Goffman, 2009).

Meanwhile, labelling and stereotyping in the early stages of stigmatization gave rise to the dimensions of negative attitudes and behaviours, namely separation and discrimination (Link & Phelan, 2006). In assessing social stigma against people with COVID-19, especially in aspects of separation and discrimination manifested in attitudes such as approval that people with COVID-19 should undergo isolation and be kept away from the community, families of COVID-19 sufferers should not live in the community. People with COVID-19 should also not get the same health services as the community.

In the social stigma against people with COVID-19, the discrimination can be a behaviour to quarantine, harassment, ostracizing, and staying away from COVID-19 sufferers and their families. Even people who have recovered from COVID-19. The stigmatizing behaviour such as reluctance to visit neighbours sick from COVID-19 and unwillingness to want to be buried with local people who have been cured of COVID-19. Other discrimination behaviours include hesitation to help funeral of COVID-19 patients and the act of not being allowed by COVID-19 to be buried in the public cemetery. The consequences of this act of discrimination will indirectly harm various aspects of survival, such as income, education, and welfare (Link & Phelan, 2006).

As previously revealed, the social stigma related to COVID-19 is very closely related to the prevention of disease transmission. Therefore, actions taken to prevent disease transmission must be fully realized by everyone so as not to turn into a stigma. If this is not done, the stigma that may arise is used as prevention, even without realizing that the action contains a high stigma. Thus, the existence of this scale of social stigma assessment becomes a tool and effort to reduce the stigma that exists.

## Conclusions

The scale of social stigma against people with COVID-19 has been declared a valid and reliable scale. This instrument can help health practitioners review and determine the social stigma in the community and how much potential stigma may occur in the community. The social stigma that can be known and measured is the first step in determining interventions to overcome the problem of COVID-19 widely. The suggestion based on the results of this study is that this instrument can be piloted back to the broader community with different cultural characteristics than the respondents in this study. Various determinant factors also need to be considered that may affect the reliability of these instruments.

## Data Availability

The datasets used and/or analyzed during the current study are available from the corresponding author on reasonable request.
